# Supplementary material for: Complement-mediated enhancement of SARS-CoV-2 antibody neutralisation potency in vaccinated individuals
Source: Nat Commun. 2025 Mar 18;16:2666. doi: 10.1038/s41467-025-57947-8 (PMC11920438; doi:10.1038/s41467-025-57947-8)
Supplement: Supplementary file 1 — Supplementary Information [file 41467_2025_57947_MOESM1_ESM.pdf]

Supplementary Information

Complement-Mediated Enhancement of SARS-CoV-2 Antibody Neutralisation Potency in Vaccinated Individuals

Authors

Jack Mellors[1*], Raman Dhaliwal[2], Stephanie Longet[3], Tom Tipton[1], OCTAVE Consortium[4***], OPTIC Consortium[4***], Eleanor Barnes[4,5], Susanna J Dunachie[4,6], Paul Klenerman[4,5], Julian Hiscox[7], Miles Carroll[1**]

Corresponding Authors

*Jack Mellors
jmellors@ic.ac.uk

**Miles Carroll
miles.carroll@ndm.ox.ac.uk

Affiliations

[1] Centre for Human Genetics and the Pandemic Sciences Institute, Nuffield Department of Medicine, University of Oxford, Oxford, United Kingdom.

[2] Sir William Dunn School of Pathology, University of Oxford, Oxford, United Kingdom

[3] Centre International de Recherche en Infectiologie, Université Jean Monnet, Université Claude Bernard Lyon, Inserm, Saint-Etienne, France

[4] NIHR Oxford Biomedical Research Centre, Oxford University Hospitals NHS Foundation Trust, Oxford, United Kingdom

[5] Translational Gastroenterology and Liver Unit, Nuffield Department of Medicine, University of Oxford, Oxford, United Kingdom

[6] NDM Centre for Global Health Research, Nuffield Department of Medicine, University of Oxford, Oxford, United Kingdom

[7] Department of Infection Biology and Microbiomes, Institute of Infection, Veterinary and Ecological Sciences, University of Liverpool, Liverpool, United Kingdom

*** A list of authors and their affiliations appears at the end of the main paper

Supplementary Figure 1: Gating strategy example for the IgG subclass assays, using OPTIC sample 4 at a 1:50 dilution for the detection of IgG2 and IgG4. Assay measures IgG1-4 binding to APC-fluorescent beads conjugated to the SARS-CoV-2 spike protein. **a** Gating of SARS-CoV-2 spike conjugated beads using FSC-A and SSC-A. **b** Gating of bead population using APC fluorescence. **c** IgG2 or **d** IgG4 measured using PE-conjugated secondary antibody. Gating and median fluorescence intensity determined using FlowJo (Version 10.10.0).

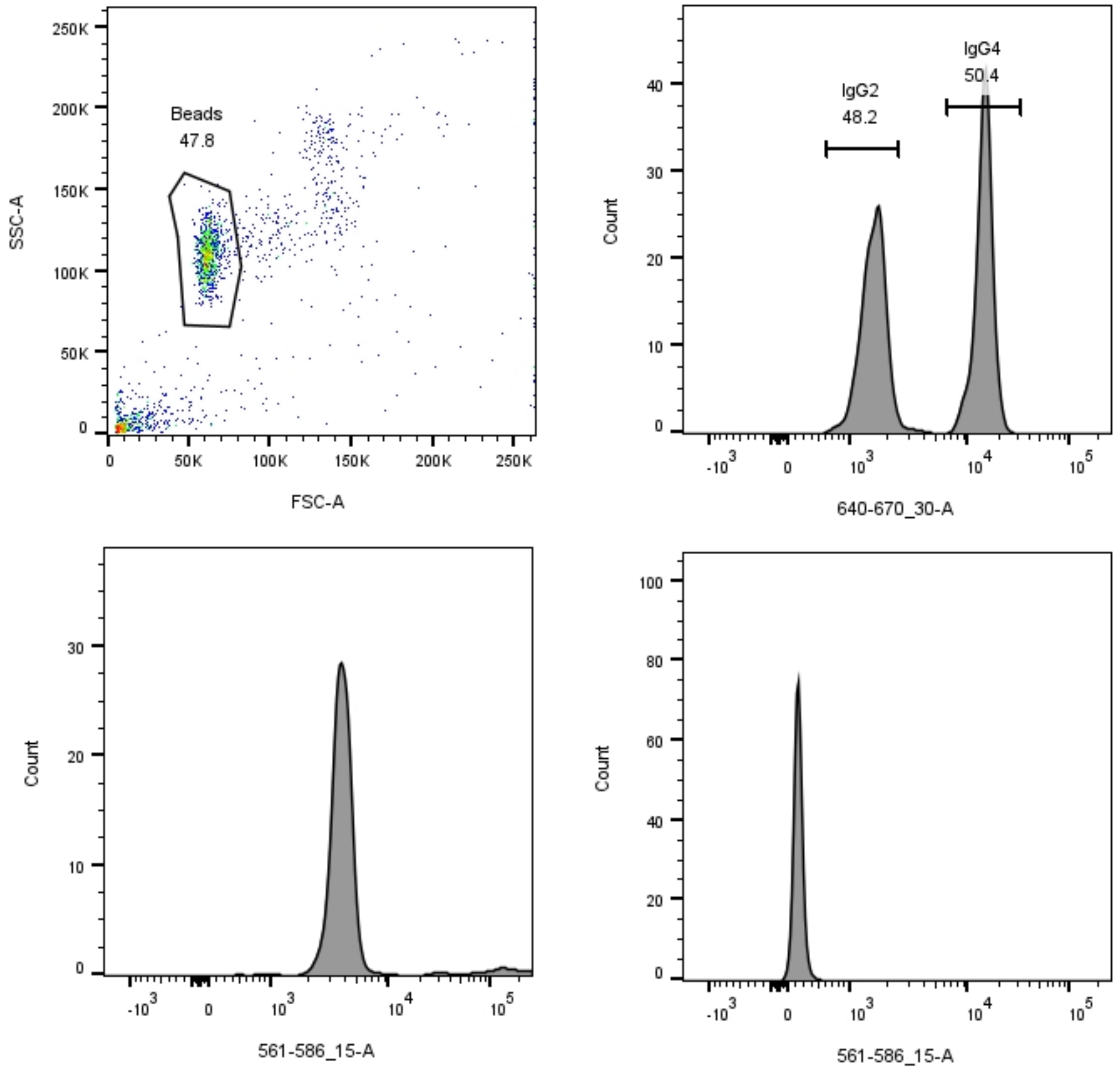

Supplementary Figure 2: Gating strategy example for antibody dependent complement deposition assays using serum from OPTIC sample 1 at a 1:50 dilution and 20% IgG/IgM-depleted complement. Assay measures C3c deposition in response to APC-fluorescent beads conjugated with the SARS-CoV-2 spike protein. **a** Gating of SARS-CoV-2 spike conjugated beads using FSC-A and SSC-A. **b** Gating of bead population using APC fluorescence. **c** Measurement of C3c deposition using FITC-conjugated antibody. Gating and median fluorescence intensity determined using FlowJo (Version 10.10.0).

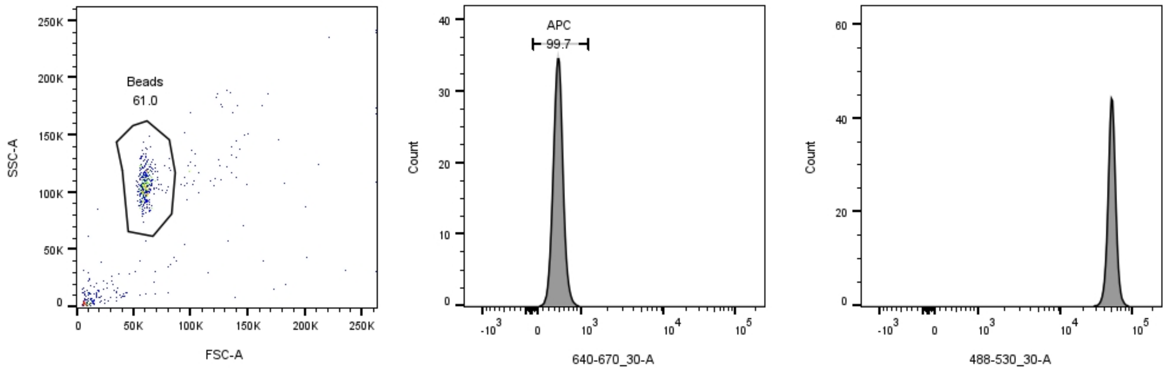

Supplementary Figure 3: Comparison of raw foci numbers as a measure of SARS-CoV-2 infection for microneutralisation assay controls in the absence of OPTIC immune sera. The addition of pooled human plasma (PHP) or heat-inactivated (HI)-FCS did not significantly reduce the level of infection in absence of immune sera. In some conditions, the level of infection was significantly higher than the conditions supplemented with media-only. Each spot indicates a single replicate from a total of 8 replicates per plate, with all plates tested. Significance with $p < 0.05$ was determined by Kruskal-Wallis test with Dunn's multiple comparisons test in GraphPad Prism (version 10). Exact *p* values for Vero E6/VIC01 (Media Vs HI-FCS, $p = < 0.0001$; Media Vs PHP, $p = < 0.0001$), Vero E6/BA.1 (Media Vs PHP, $p = < 0.0001$, HI-FCS Vs PHP, $p = < 0.0001$), Calu-3/VIC01 (Media Vs HI-FCS, $p = 0.0049$), Calu-3/BA.1 (Media Vs HI-FCS, $p = < 0.0001$; Media Vs PHP, $p = < 0.0001$; HI-FCS Vs PHP, $p = 0.0020$). * $p < 0.05$, ** $p < 0.01$, *** $p < 0.001$, **** $p < 0.0001$. Source data are provided as a Source Data file.

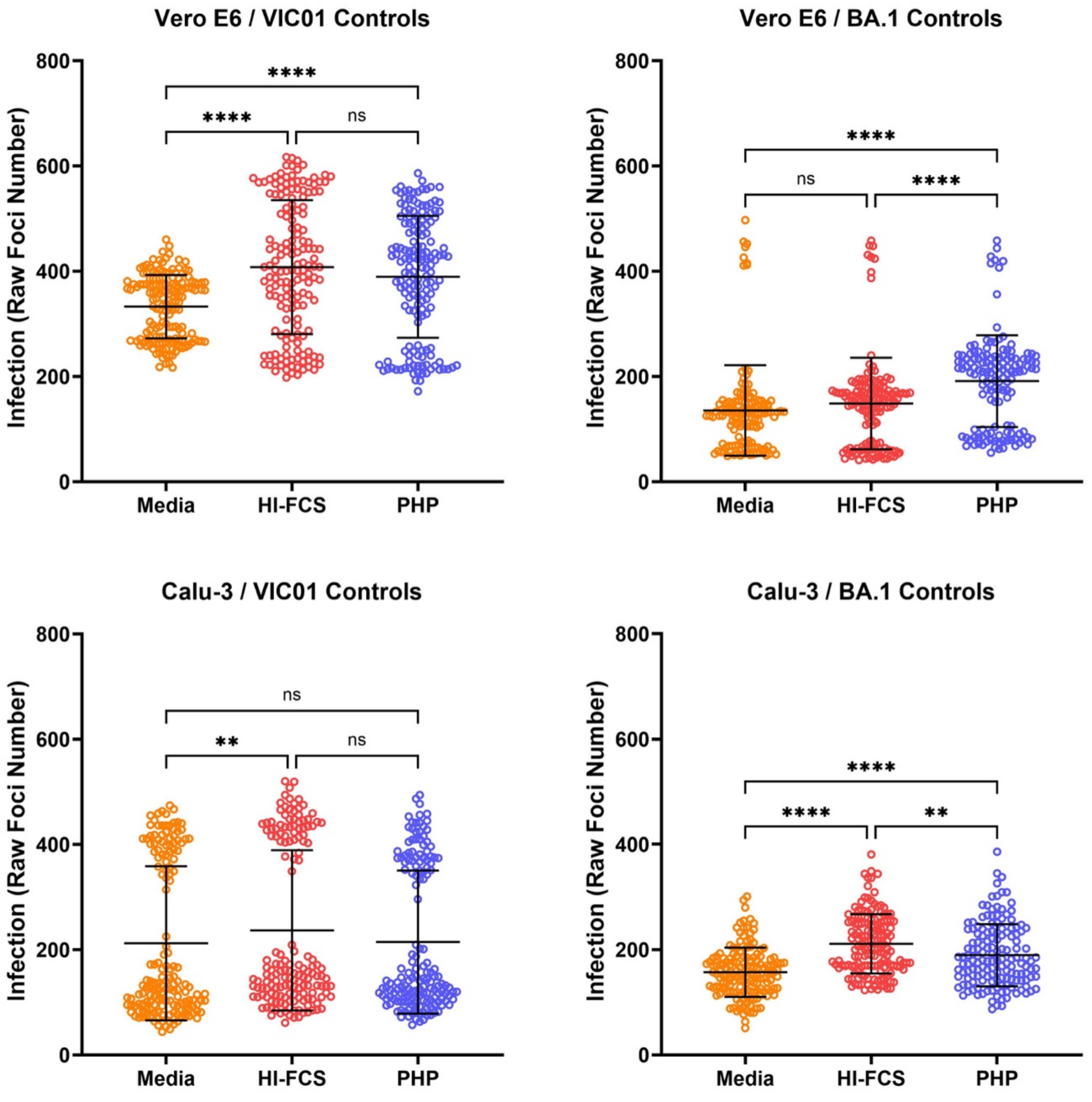

Supplementary Figure 4: Crystal violet staining of complement-only conditions to measure cytotoxicity. **(a)** Cytotoxicity test using Calu-3 cells with media only, 20% pooled human plasma (PHP), 20% C3/C5 depleted plasma, or 1.8% DMSO as a positive control. Tearing from manual pipetting was evident in some conditions and cytotoxicity was evident only in the 1.8% DMSO condition. **(b)** Cytotoxicity test using Vero E6 cells with media only, 20% PHP, or 3% DMSO as a positive control. Conditions replicated the microneutralisation assay procedure and cells were stained with 0.2% crystal violet in 20% ethanol. Cytotoxicity was only evident in the 3% DMSO condition.

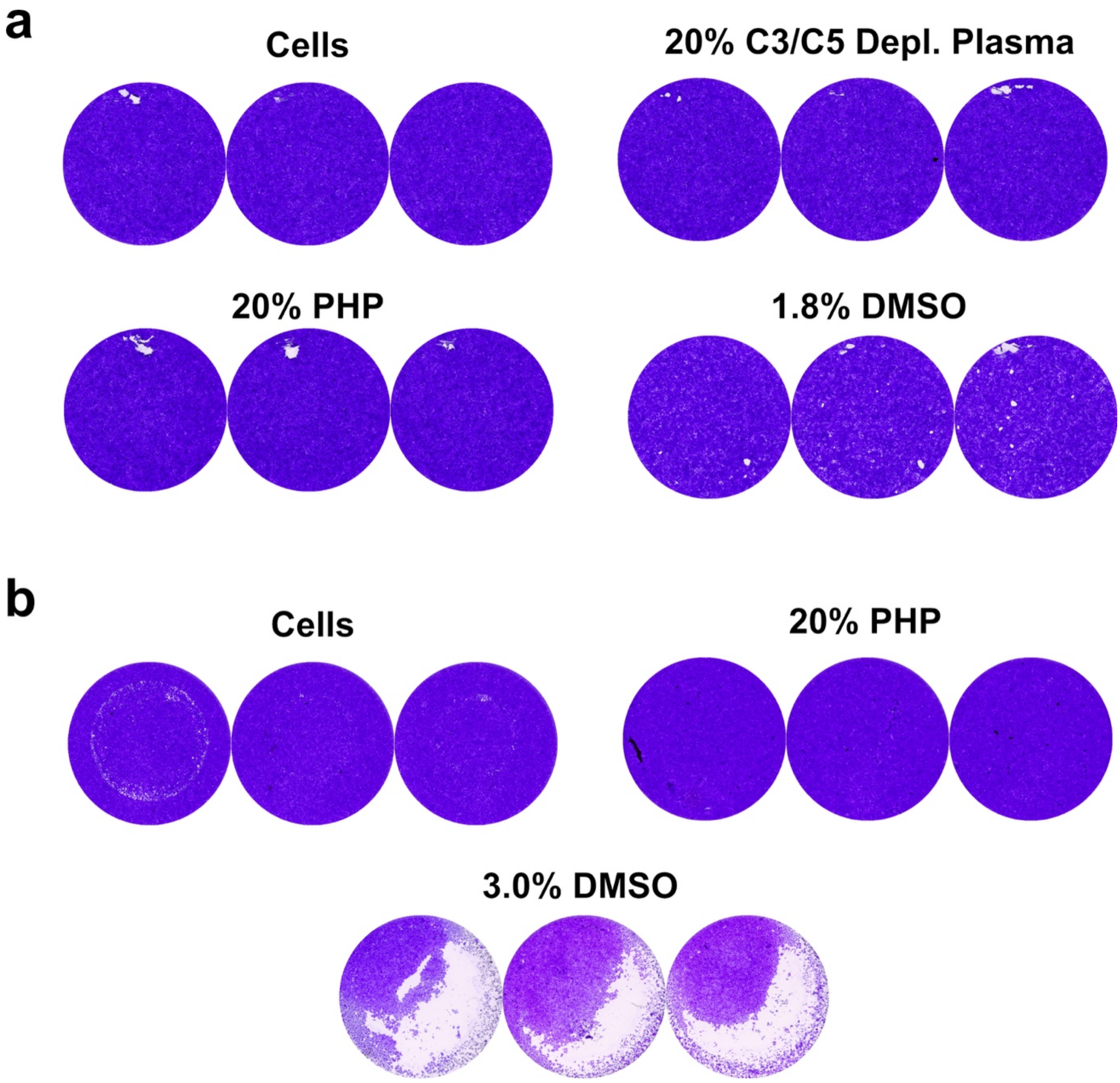

Supplementary Figure 5: Transmission electron microscopy images of SARS-CoV-2 in the presence of OPTIC serum sample 10 and pooled human plasma (PHP). No clear difference was observed between the virus-only condition and the addition of immune sera with PHP or heat-inactivated PHP (HI-PHP). The black arrows indicate examples of SARS-CoV-2 particles.

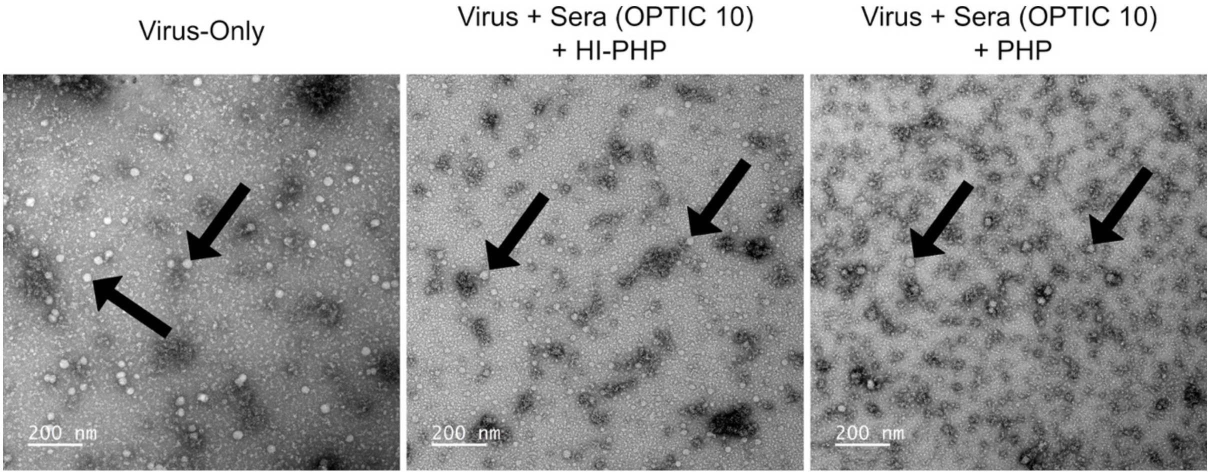

Supplementary Figure 6: Pearson correlations comparing IgG binding to Coronavirus spike proteins (determined via Meso Scale Discovery) against neutralisation for the Enhanced Group (*n* = 14) and Non-Enhanced Group (*n* = 17). Corrected for multiple comparisons with Benjamini Hochberg false discovery rate of 0.05 in GraphPad Prism (version 10). * p <0.05, ** p < 0.01, *** p < 0.001, **** p < 0.0001. Nucleocapsid (N); pooled human plasma (PHP); receptor binding domain (RBD); spike (S). Source data are provided as a Source Data file.

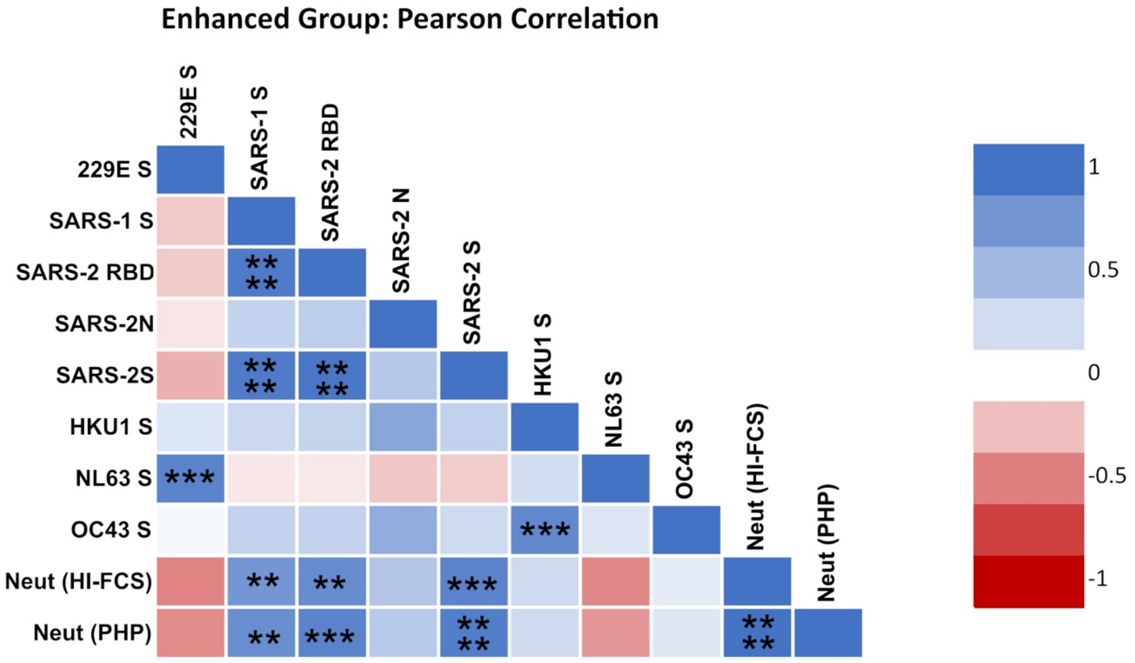

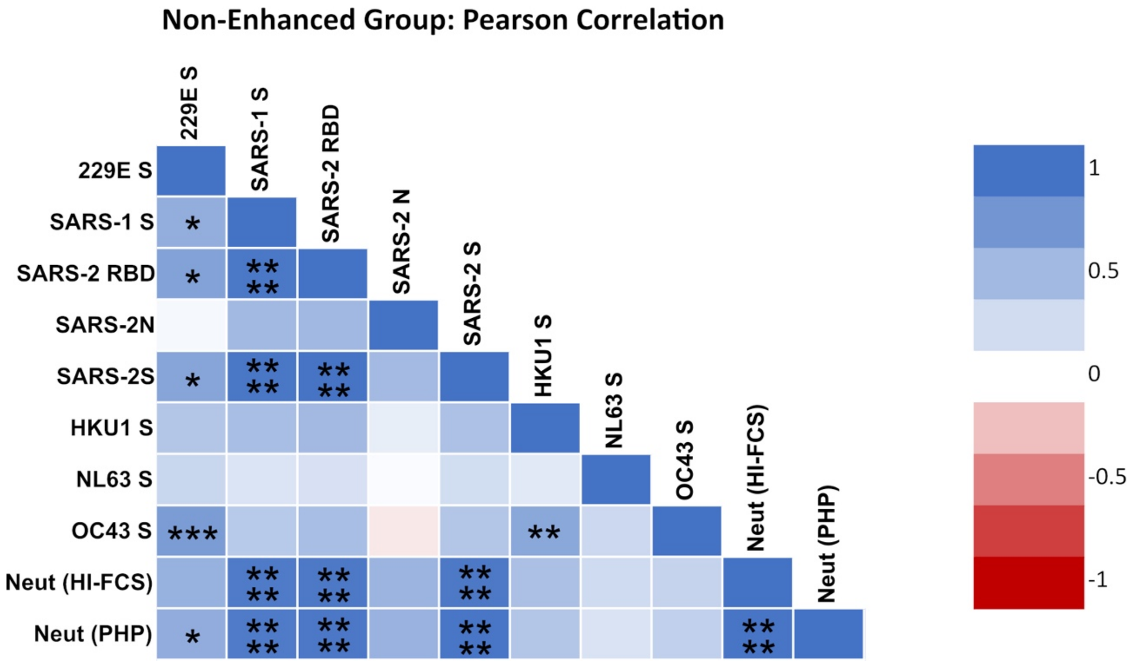

Supplementary Figure 7: Mean decrease in accuracy and receiver operating characteristic (ROC) curve for random forest analysis to determine complement-mediated enhancement of neutralisation (*n* = 30). Analysis was performed in R Studio using the ‘randomForest’ and ‘pROC’ packages.

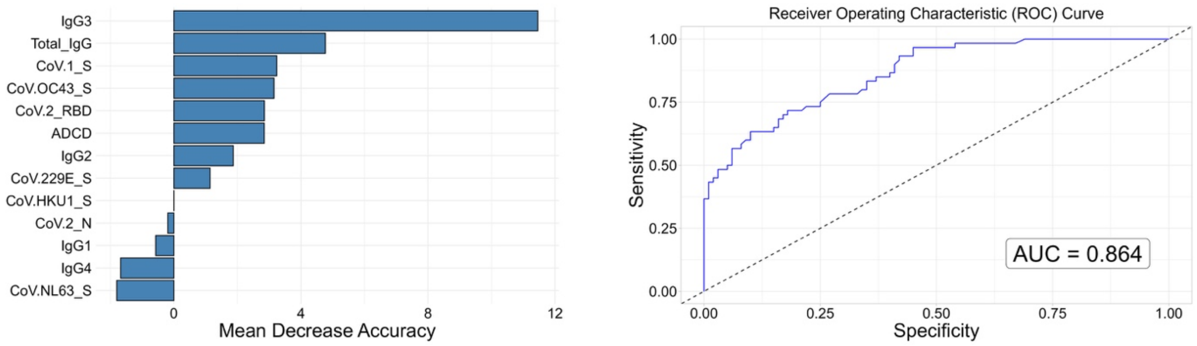

Supplementary Figure 8: SARS-CoV-2 neutralisation titres comparing the use of Vero E6 cells with and without TMPRSS2 expression. Normalised neutralisation titres supplemented with heat-inactivated pooled human plasma (PHP) were subtracted from values supplemented with PHP to determine the increase in complement-mediated neutralisation. Error bars show the mean and standard deviation. Results were non-significant (p = 0.274) as determined by a paired, two-tailed t test where p < 0.05 is significant. Source data are provided as a Source Data file.

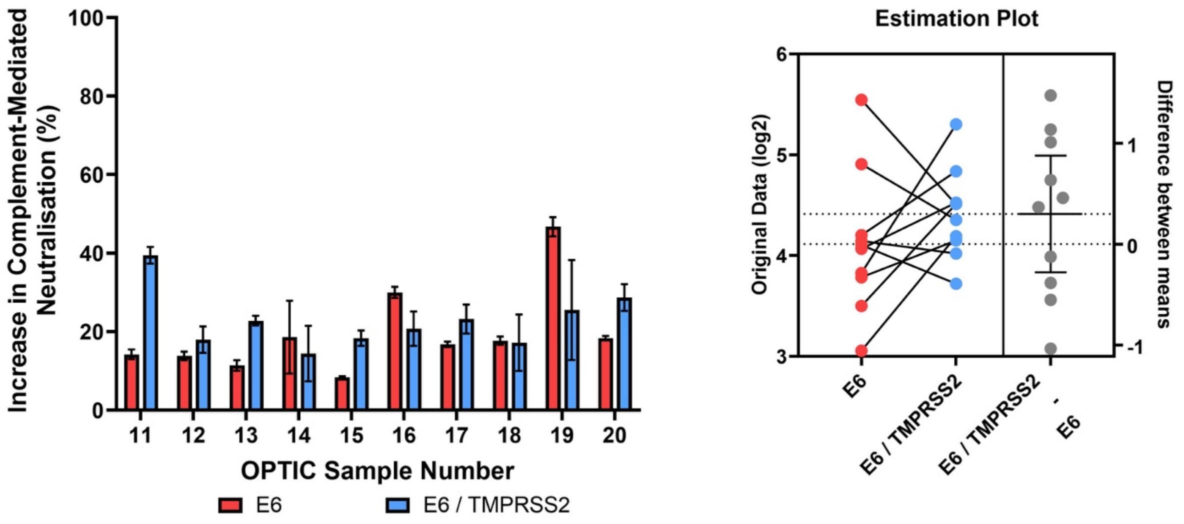

Supplementary Figure 9: Correlations with IgM and IgG binding to Coronavirus spike proteins determined via Meso Scale Discovery (MSD) against SARS-CoV-2 (VIC01 strain) neutralisation titres for the OCTAVE (n = 21) and OPTIC (n = 10) cohorts. Corrected for multiple comparisons using Benjamini Hochberg false discovery rate of 0.05. Statistical analysis was performed in GraphPad Prism (Version 10). * $p < 0.05$, ** $p < 0.01$, *** $p < 0.001$, **** $p < 0.0001$. Nucleocapsid (N); pooled human plasma (PHP); receptor binding domain (RBD); spike (S). Source data are provided as a Source Data file.

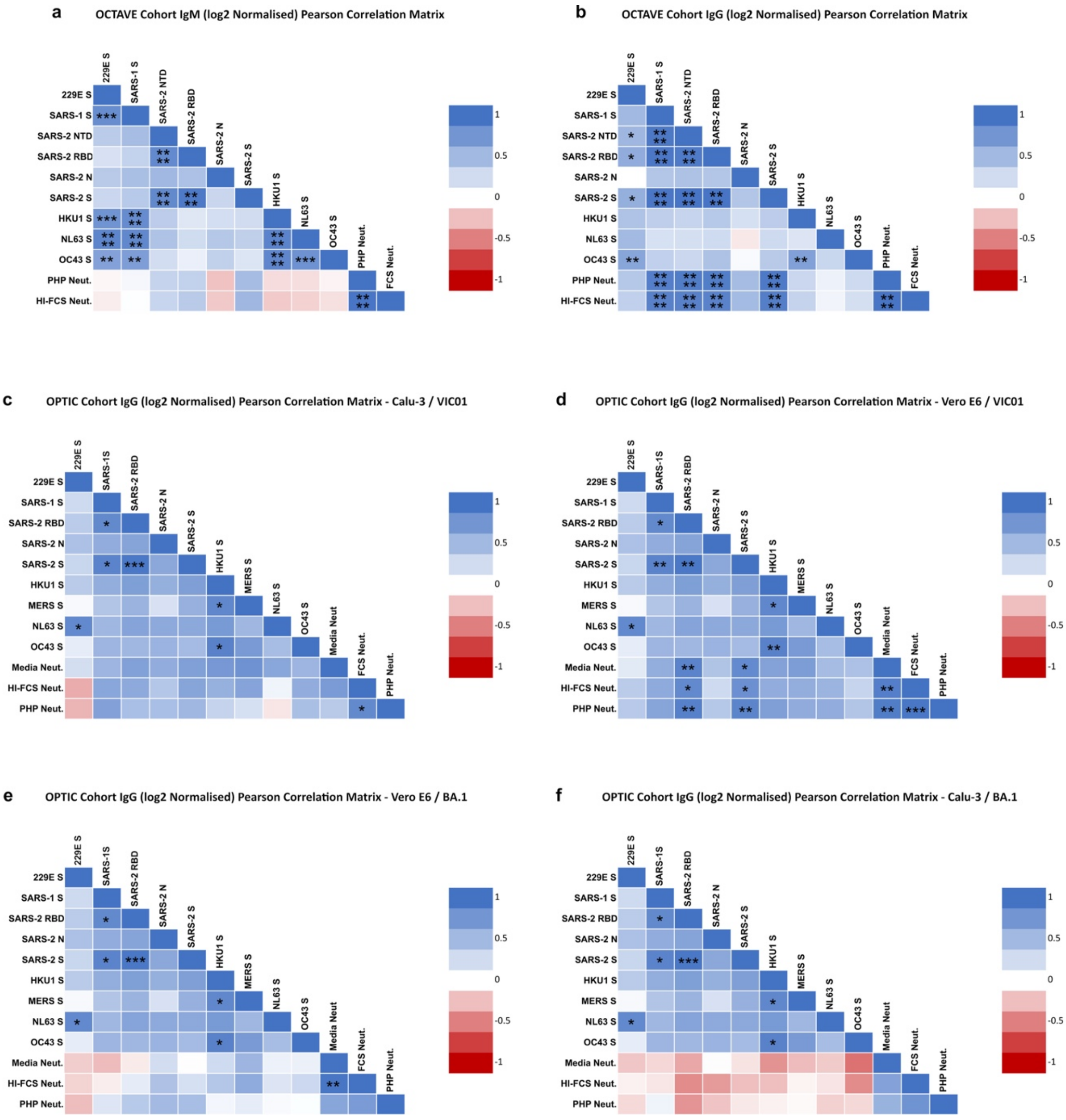

Supplementary Figure 10: Comparison of antibody characteristics between the OPTIC and OCTAVE cohorts. **a** Total SARS-CoV-2 spike IgG titres measured via electrochemiluminescence (ECL) (OPTIC, *n* = 10; OCTAVE, *n* = 21). **b** Median fluorescence intensity (MFI) of IgG1-4 in all samples (OPTIC, *n* = 9; OCTAVE, *n* = 21). **c** Pairwise comparison of MFI of IgG1-4 SARS-CoV-2 spike specific titres (OPTIC, *n* = 9; OCTAVE, *n* = 21). **d** Pairwise comparison of antibody-dependent complement deposition (ADCD) between the two cohorts (OPTIC, *n* = 9; OCTAVE, *n* = 21). Statistical significance for **a – d** was determined via an unpaired, two-tailed t test in GraphPad Prism (Version 10). Error bars show the mean value with standard deviation. **e** Pearson correlation with Benjamini Hochberg false discovery rate of 0.05 to compare relationships of antibody characteristics within the two cohorts. * $p < 0.05$, ** $p < 0.01$, *** $p < 0.001$, **** $p < 0.0001$. Source data are provided as a Source Data file.

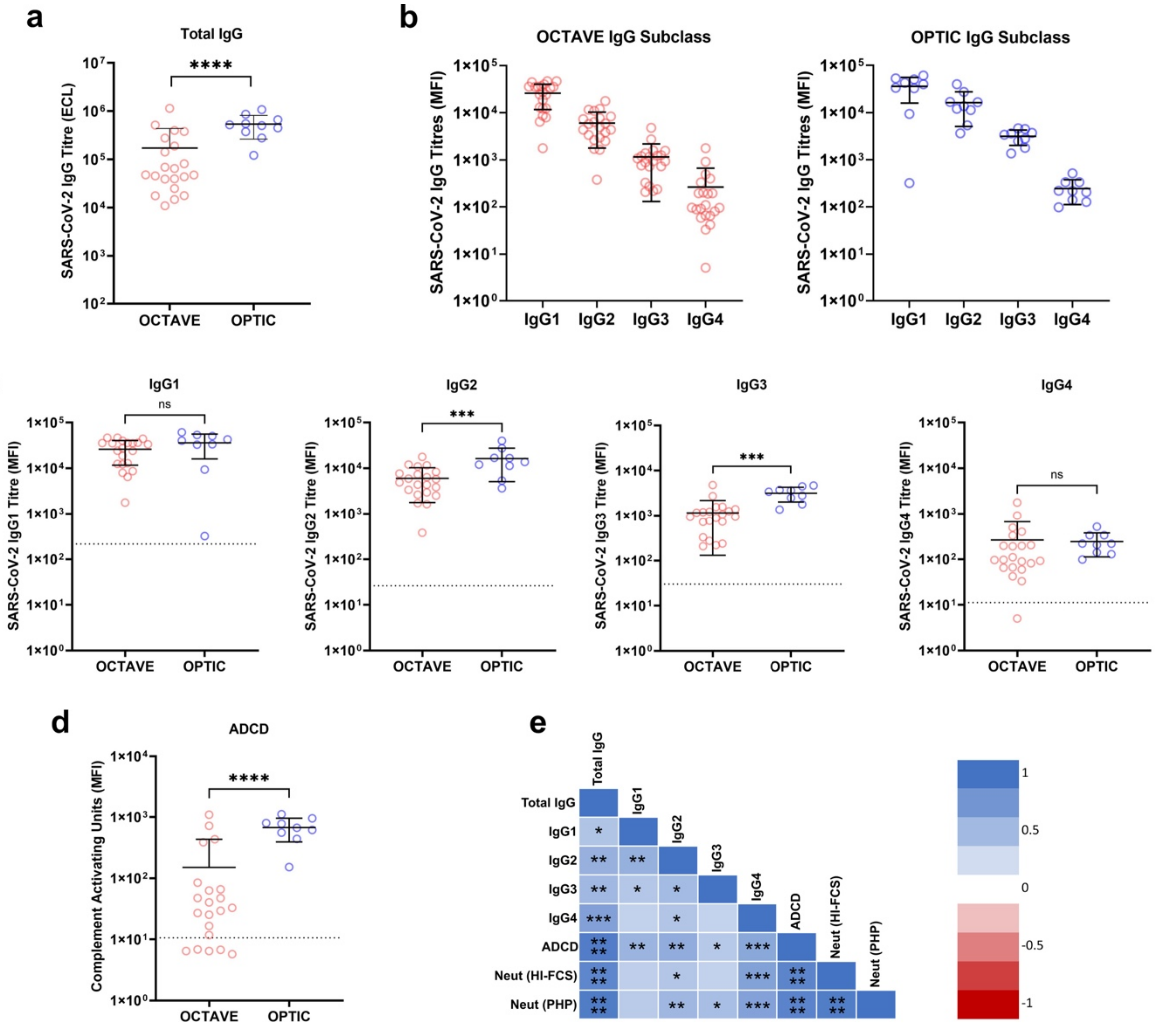

Supplementary Table 1: Meso Scale Discovery (MSD) SARS-CoV-2 antigen information for ACE2 competition assays. Information supplied by MSD as reported in the product documentation “V-PLEX COVID-19 ACE2 Neutralization Assays insert”.

Spot	SARS-CoV-2 Antigen	Lineage	Amino Acid Modifications
1	Spike (Wuhan)	Wuhan	Not Applicable
2	Spike (BA.2.12.1)	Omicron	T19I, (L24-A27)toS, G142D, V213G, G339D, S371F, S373P, S375F, T376A, D405N, R408S, K417N, N440K, L452Q, S477N, T478K, E484A, Q493R, Q498R, N501Y, Y505H, D614G, H655Y, N679K, P681H, S704LN764K, D796Y, Q954H, N969K
3	Nucleocapsid	Wuhan	Not Applicable
4	Spike (BA.2.75)	Omicron	T19I, L24-A27>S, G142D, K147E, W152R, F157L, I210V, V213G, G257S, G339H, S371F, S373P, S375F, T376A, D405N, R408S, K417N, N440K, G446S, N460K, S477N, T478K, E484A, Q498R, N501Y, Y505H, D614G, H655Y, N679K, P681H, N764K, D796Y, Q954H, N969K
5	Spike (BA.2; BA.2.1; BA.2.2; BA.2.3; BA.2.5; BA.2.6; BA.2.7; BA.2.8; BA.2.10; BA.2.12)	Omicron	T19I, (L24-A27)toS, G142D, V213G, G339D, S371F, S373P, S375F, T376A, D405N, R408S, K417N, N440K, S477N, T478K, E484A, Q493R, Q498R, N501Y, Y505H, D614G, H655Y, N679K, P681H, N764K, D796Y, Q954H, N969K
6	Spike (B.1.1.529; BA.1; BA.1.15)	Omicron	A67V, ΔH69-V70, T95I, G142D, Δ143-145, Δ211/L212I, ins214EPE, G339D, S371L, S373P, S375F, K417N, N440K, G446S, S477N, T478K, E484A, Q493R, G496S, Q498R, N501Y, Y505H, T547K, D614G, H655Y, N679K, P681H, N764K, D796Y, N856K, Q954H, N969K, L981F
7	Spike (B.1.617.2; AY.4)	Delta	**(Alt Seq 2):** T19R, T95I, G142D, Δ156/157, R158G, L452R, T478K, D614G, P681R, D950N
8	Spike (B.1.1.7)	Alpha	ΔH69-V70, ΔY144, N501Y, A570D, D614G, P681H, T716I, S982A, D1118H
9	Spike (B.1.351)	Beta	L18F, D80A, D215G, Δ242-244, R246I, K417N, E484K, N501Y, D614G, A701V
10	Spike (BA.5)	Omicron	T19I, (L24-A27)toS, del69/70, G142D, V213G, G339D, S371F, S373P, S375F, T376A, D405N, R408S, K417N, N440K, L452R, S477N, T478K, E484A, F486V, Q498R, N501Y, Y505H, D614G, H655Y, N679K, P681H, N764K, D796Y, Q954H, N969K

Note: Alternative S-GENE mutations for Spike of B.1.617.2 is listed as "Alt Seq 2."
