# Supplementary material for: Complement-mediated enhancement of SARS-CoV-2 antibody neutralisation potency in vaccinated individuals
Source: Nat Commun. 2025 Mar 18;16:2666. doi: 10.1038/s41467-025-57947-8 (PMC11920438; doi:10.1038/s41467-025-57947-8)
Supplement: Supplementary file 2 — Description of Additional Supplementary Files [file 41467_2025_57947_MOESM2_ESM.pdf]

Description of Additional Supplementary Files

File Name: Supplementary Data 1
Description: All NT50 values for the OPTIC cohort with individual values and significance

File Name: Supplementary Data 2
Description: All NT50 values for the OCTAVE cohort with individual values and significance
