# Supplementary material for: Complement-mediated enhancement of SARS-CoV-2 antibody neutralisation potency in vaccinated individuals
Source: Nat Commun. 2025 Mar 18;16:2666. doi: 10.1038/s41467-025-57947-8 (PMC11920438; doi:10.1038/s41467-025-57947-8)
Supplement: Supplementary file 5 — Reporting Summary [file 41467_2025_57947_MOESM5_ESM.pdf]

nature portfolio

Corresponding author(s): Jack Mellors
Miles Carroll

Last updated by author(s): Nov 5, 2024

Reporting Summary

Nature Portfolio wishes to improve the reproducibility of the work that we publish. This form provides structure for consistency and transparency in reporting. For further information on Nature Portfolio policies, see our Editorial Policies and the Editorial Policy Checklist.

Statistics

For all statistical analyses, confirm that the following items are present in the figure legend, table legend, main text, or Methods section.

n/a	Confirmed	
☐	☒	The exact sample size (*n*) for each experimental group/condition, given as a discrete number and unit of measurement
☐	☒	A statement on whether measurements were taken from distinct samples or whether the same sample was measured repeatedly
☐	☒	The statistical test(s) used AND whether they are one- or two-sided<br>*Only common tests should be described solely by name; describe more complex techniques in the Methods section.*
☐	☒	A description of all covariates tested
☐	☒	A description of any assumptions or corrections, such as tests of normality and adjustment for multiple comparisons
☐	☒	A full description of the statistical parameters including central tendency (e.g. means) or other basic estimates (e.g. regression coefficient) AND variation (e.g. standard deviation) or associated estimates of uncertainty (e.g. confidence intervals)
☐	☒	For null hypothesis testing, the test statistic (e.g. *F*, *t*, *r*) with confidence intervals, effect sizes, degrees of freedom and *P* value noted<br>*Give P values as exact values whenever suitable.*
☒	☐	For Bayesian analysis, information on the choice of priors and Markov chain Monte Carlo settings
☐	☒	For hierarchical and complex designs, identification of the appropriate level for tests and full reporting of outcomes
☐	☒	Estimates of effect sizes (e.g. Cohen's *d*, Pearson's *r*), indicating how they were calculated

Our web collection on statistics for biologists contains articles on many of the points above.

Software and code

Policy information about availability of computer code

Data collection	The code used within this manuscript only used data generated within this manuscript or in previous publications which are then made accessible through this manuscript. Sample code and input data for the analyses in this study are available on GitHub: https://github.com/jmellors/Complement-Mediated-Enhancement-of-SARS-CoV-2-Antibody-Neutralisation-Potency. DOI: 10.5281/zenodo.14548585
Data analysis	All statistical analyses were performed in GraphPad Prism (Version 10) where p < 0.05 was considered significant. Normality tests were performed on all samples prior to analysis. Random forest and logistic regression with LASSO and ridge methods were performed in R/R Studio (version 4.4.1).<br><br>Random forest was performed using the R package 'randomForest' with 500 trees and 4 variables at each split. The dataset was split 70/30 with training and test data respectively, and performed across 20 iterations. The Least Absolute Shrinkage and Selection Operator (LASSO) and ridge logistic regression was performed using the R package 'glmnet', with alpha = 1 (LASSO) or alpha = 0 (ridge) and the optimal regularisation parameter lambda was determined through 10-fold cross-validation. This was followed by bootstrapping (B = 1000) to generate 95% confidence intervals and variables with CIs non-overlapping zero were considered important. Important variables for LASSO regression were non-zero coefficients.

For manuscripts utilizing custom algorithms or software that are central to the research but not yet described in published literature, software must be made available to editors and reviewers. We strongly encourage code deposition in a community repository (e.g. GitHub). See the Nature Portfolio guidelines for submitting code & software for further information.

Data

Policy information about availability of data

All manuscripts must include a data availability statement. This statement should provide the following information, where applicable:

- Accession codes, unique identifiers, or web links for publicly available datasets
- A description of any restrictions on data availability
- For clinical datasets or third party data, please ensure that the statement adheres to our policy

The authors declare that the data supporting the findings of this study are available within the paper and its supplementary information files. All data generated within this study is provided in the Source Data file.

Research involving human participants, their data, or biological material

Policy information about studies with human participants or human data. See also policy information about sex, gender (identity/presentation), and sexual orientation and race, ethnicity and racism.

Reporting on sex and gender	No sex- and gender-based analyses have been performed. This study utilised residual samples from other studies where complete matching based on age, sex, gender, ethnicity etc was not feasible. This does not impact the conclusion made regarding the paper nor do we believe it relevant to the analysis conducted or the topic of study. It has been explicitly stated within the manuscript that samples were not matched based on these conditions and that conclusion are made within these parameters. Sex was determined from self-reporting and the information can be made available upon request.
Reporting on race, ethnicity, or other socially relevant groupings	No race and ethnicity based analyses have been performed. This study utilised residual samples from other studies where complete matching based on race and ethnicity was not feasible. This does not impact the conclusion made regarding the paper nor do we believe it relevant to the analysis conducted or the topic of study. It has been explicitly stated within the manuscript that samples were not matched based on these conditions and that conclusion are made within this parameters.
Population characteristics	The serum from individuals in the OCTAVE cohort used within this study (n = 21) were collected 25 – 67 days post-boost with the ChAdOx1 Vaccine, between May – July 2021. These samples were randomly selected and the corresponding individuals had a diagnosis belonging to one of the following groups: autoimmune hepatitis (n = 2), liver cirrhosis (Child Pugh A (n = 6) or Child Pugh B (n = 3)), Crohn's disease (n = 2), ulcerative colitis (n = 6), kidney transplant (n = 2).<br><br>The serum from individuals in the OPTIC cohort used within this study (n = 10) were collected 7-days post-boost after vaccination with the COVID-19 mRNA Vaccine BNT162b2 (Pfizer), in January 2021. The samples were randomly selected for use in this study. Age and genotypic information where therefore not included for this particular study.
Recruitment	The samples utilised in this study were residual samples from other ongoing studies.<br>The OCTAVE (Observational Cohort trial T cells, Antibodies and Vaccine Efficacy in SARS-CoV-2) trial (ISRCTN 12821688) aims to assess the SARS-CoV-2 vaccine responses of immunocompromised individuals that were part of the UK national COVID-19 vaccination programme and the majority of subjects received either the COVID-19 mRNA vaccine BNT162b2 (Pfizer/BioNTech) or the ChAdOx1 Vaccine (AstraZeneca formerly AZD1222).<br><br>The OPTIC (Oxford Protective T-cell Immunity to Coronavirus) study is a prospective, longitudinal observational cohort study of healthcare workers (HCWs) as part of the national PITCH (Protective Immunity from T Cells in Healthcare workers) consortium. HCWs defined as SARS-CoV-2 naïve based on documented PCR and/or serology results were recruited after vaccination with the COVID-19 mRNA Vaccine BNT162b2 (Pfizer).
Ethics oversight	The OCTAVE Trial was approved by the UK Medicines and Healthcare Products Regulatory Agency (MHRA) on 5 February 2021 and by the London and Chelsea Research Ethics Committee (REC ref.: 21/HRA/0489) on 12 February 2021. The protocol has subsequently been amended eight times with five substantial amendments (with ethical approvals dated 3 March 2021, 19 April 2021, 24 December 2021 and 4 April 2022) and three non-substantial amendments: protocol versions dated 22 April 2021, 14 July 2021 and 10 September 2021. The trial is registered on ISRCTN12821688. The OPTIC healthcare worker participants were recruited under the GI Biobank Study 16/YH/0247, approved by the research ethics committee (REC) at Yorkshire & The Humber - Sheffield Research Ethics Committee on 29 July 2016, which was amended for this purpose on 8 June 2020. All patients and participants provided their written informed consent to participate in this study.

Note that full information on the approval of the study protocol must also be provided in the manuscript.

Field-specific reporting

Please select the one below that is the best fit for your research. If you are not sure, read the appropriate sections before making your selection.

☒ Life sciences ☐ Behavioural & social sciences ☐ Ecological, evolutionary & environmental sciences

For a reference copy of the document with all sections, see nature.com/documents/nr-reporting-summary-flat.pdf

Life sciences study design

All studies must disclose on these points even when the disclosure is negative.

Sample size	This study used a total sample size of 31 samples (10 from OPTIC cohort and 21 from OCTAVE cohort). This study could in theory have been conducted using a few purified monoclonal antibodies and so we believe a samples size of 31 is sufficient for the conclusions made. It also provides statistical power to perform comparisons within and between populations.
Data exclusions	No data was excluded within this study.
Replication	It is explicitly stated within the manuscript for each experiments where technical replicates were performed either in duplicate or triplicate, and experimental replicates were also conducted either in duplicate or in triplicate. All attempts at replication were successful and are the data provided within the source data.
Randomization	Samples were already part of UK vaccination trials as previously described and the same allocation was used within this study. Samples were selected based on availability of serum. Any subsequent grouping made within the study was done so based on the findings within this study i.e. whether samples showed a significant enhancement of neutralisation in the presence of complement.
Blinding	Blinding was not relevant to the study as no hypothesis was made regarding the differences between groups or what they might be. The only available information during the time of the study was which samples belonged to which group. Whilst two individual groups were tested, this was simply the utilisation of pre-existing samples from other studies and was not important for this study.

Reporting for specific materials, systems and methods

We require information from authors about some types of materials, experimental systems and methods used in many studies. Here, indicate whether each material, system or method listed is relevant to your study. If you are not sure if a list item applies to your research, read the appropriate section before selecting a response.

Materials & experimental systems

n/a	Involved in the study	
	x	Antibodies
	x	Eukaryotic cell lines
x		Palaeontology and archaeology
x		Animals and other organisms
x		Clinical data
	x	Dual use research of concern
x		Plants

Methods

n/a	Involved in the study	
x		ChIP-seq
	x	Flow cytometry
x		MRI-based neuroimaging

Antibodies

Antibodies used

Anti-SARS-CoV-2 nucleocapsid - provided by Tiong Tan at the Radcliffe Department of Medicine, University of Oxford, UK.
Concentration: 1:4000 dilution

Peroxidase-conjugated Anti-human IgG - Merck - cat no: A0170-1ML - Polyclonal
Concentration: 1:5000 dilution

PE-conjugated Anti-human IgG1 - Cambridge Bioscience - cat no: 9052-09 - Clone: 4E3
Concentration: 1μg/ml

PE-conjugated Anti-human IgG2 - Cambridge Bioscience - cat no: 9060-09 - Clone: 31-7-4
Concentration: 1μg/ml

PE-conjugated Anti-human IgG3 - Cambridge Bioscience - cat no: 9210-09 - Clone: HP6050
Concentration: 1μg/ml

PE-conjugated Anti-human IgG4 - Cambridge Bioscience - cat no: 9200-09 - Clone: HP6025
Concentration: 1μg/ml

FITC-conjugated C3c antibody - Abcam - cat no: ab4212 - Polyclonal
Concentration: 1:500 dilution

Validation

All antibodies were validated through the data provided in the manuscript. This included the use of all relevant positive and negative controls alongside all test samples. Antibody performance was also verified in other relevant citations:

Peroxidase-conjugated Anti-human IgG - Merck - cat no: A0170-1ML
Cited in >20 publications. https://www.sigmaaldrich.com/GB/en/product/sigma/a0170

PE-conjugated Anti-human IgG1 - Cambridge Bioscience - cat no: 9052-09
Cited in 21 publications. https://www.bioscience.co.uk/product~142939

PE-conjugated Anti-human IgG2 - Cambridge Bioscience - cat no: 9060-09
Cited in 22 publications. https://resources.southernbiotech.com/techbul/9060.pdf

PE-conjugated Anti-human IgG3 - Cambridge Bioscience - cat no: 9210-09
Cited in 22 publications. https://www.bioscience.co.uk/product~142995

PE-conjugated Anti-human IgG4 - Cambridge Bioscience - cat no: 9200-09
Cited in 22 publications. https://www.bioscience.co.uk/product~142989

FITC-conjugated C3c antibody - Abcam - cat no: ab4212
Suitable for ICC/IF and reacts with Human samples. Cited in 10 publications. Immunogen corresponding to Native Full Length Protein corresponding to Human C3. https://www.abcam.com/en-us/products/primary-antibodies/fitc-c3c-antibody-ab4212#

Eukaryotic cell lines

Policy information about cell lines and Sex and Gender in Research

Cell line source(s)	Vero E6 - European Culture of Authenticated Cell Cultures (non-human primate kidney, Vero 76, clone E6, European Culture of Authenticated Cell Cultures, Salisbury, UK, 85020206)<br>Vero E6 with TMPRSS2 - NIBSC Research Reagent Repository, UK. NIBSC Reference 100978<br>Calu-3 - American Type Culture Collection (human lung adenocarcinoma, ATCC, HTB-55)
Authentication	Authentication performed by reference laboratories from which the cell lines were obtained.
Mycoplasma contamination	All cell lines tested negative for mycoplasma contamination
Commonly misidentified lines (See ICLAC register)	No commonly misidentified cell lines were used in the study.

Dual use research of concern

Policy information about [dual use research of concern]

Hazards

Could the accidental, deliberate or reckless misuse of agents or technologies generated in the work, or the application of information presented in the manuscript, pose a threat to:

No	Yes	
☒	☐	Public health
☒	☐	National security
☒	☐	Crops and/or livestock
☒	☐	Ecosystems
☒	☐	Any other significant area

Experiments of concern

Does the work involve any of these experiments of concern:

No	Yes	
☒	☐	Demonstrate how to render a vaccine ineffective
☒	☐	Confer resistance to therapeutically useful antibiotics or antiviral agents
☒	☐	Enhance the virulence of a pathogen or render a nonpathogen virulent
☒	☐	Increase transmissibility of a pathogen
☒	☐	Alter the host range of a pathogen
☒	☐	Enable evasion of diagnostic/detection modalities
☒	☐	Enable the weaponization of a biological agent or toxin
☒	☐	Any other potentially harmful combination of experiments and agents

Plants

Seed stocks	NA
Novel plant genotypes	NA
Authentication	NA

Flow Cytometry

Plots

Confirm that:

☒ The axis labels state the marker and fluorochrome used (e.g. CD4-FITC).

☒ The axis scales are clearly visible. Include numbers along axes only for bottom left plot of group (a 'group' is an analysis of identical markers).

☒ All plots are contour plots with outliers or pseudocolor plots.

☒ A numerical value for number of cells or percentage (with statistics) is provided.

Methodology

Sample preparation	Bead Preparation<br>To determine the IgG subclasses and ADCD of the OPTIC and OCTAVE serum samples, APC-fluorescent beads conjugated to the SARS-CoV-2 whole spike protein were used as previously described. 500 µl of SPHEROTM Magnetic Flow Cytometry Multiplex Bead Assay particles (Spherotech) were pelleted using the EasyEightsTM EasySepTM Magnet (STEMCELL

Technologies), washed in 82mM sodium phosphate buffer (pH 6.2), and activated in the same buffer containing 1.24 mg of N-hydroxysulfosuccinimide and 1-ethyl-3-[3-dimethlyyaminopropyl]carbodiimide-HCl) for 20 min. The beads were then pelleted and washed twice in coupling buffer (50mM 2-(N-morpholino) ethanesulfonic acid, pH 5.0) and resuspended in coupling buffer containing 14.5 µg of SARS-CoV-2 spike protein from the ancestral strain (Lake Pharma, 46328) for 2 hrs on a rotational mixer. The conjugated beads were then pelleted and washed twice in blocking buffer (PBS containing 2% BSA and 0.05% sodium azide, pH 7.4) and resuspended in the same buffer overnight on a rotational mixer. The beads were then pelleted, washed and resuspended in 500 µl of PBS containing 0.05% sodium azide and stored at 4oC until use.

IgG Subclass Assay
SARS-CoV-2-conjugated magnetic, fluorescent beads were prepared as previously described and used to determine the levels of IgG1, IgG2, IgG3, and IgG4 in the OPTIC and OCTAVE serum samples. The beads were diluted to a concentration of 50 beads/µl and 20 µl of the diluted beads was added to each well with 30 µl of PBS and heat-inactivated serum at a final dilution of 1:50, conducted in duplicate. The beads and serum were incubated for 1 hr at RT whilst shaking at 700 rpm, then washed twice in 100 µl of wash buffer (PBS containing 0.1% tween-20) and resuspended in 100 µl of 1µg/ml PE-conjugated IgG1 (Cambridge Bioscience), IgG2 (Cambridge Bioscience), IgG3 (Cambridge Bioscience), or IgG4 (Cambridge Bioscience) antibody in PBS. The samples were incubated for 1 hr at RT whilst shaking at 700 rpm and then washed twice in 100 µl of wash buffer before resuspending in 50 µl of PBS. Duplicate samples were combined and a minimum of 100 beads per sample were acquired on the BD LSRFortessa X-20 flow cytometer. The mean fluorescence intensity of PE was determined using FlowJo (version 10) with the gating strategy shown in Supplementary Figure 1. The background fluorescence for each serum sample was measured in the absence of secondary antibody and subtracted from the raw MFI values. A quality control sample was included in each experiment for all IgG subclasses to ensure reproducibility. A SARS-CoV-2 IgG negative sample was also included in each experiment for all IgG subclasses where the mean MFI plus three standard deviations across all replicates was used to determine the limit of detection.

ADCD Assay
SARS-CoV-2-conjugated magnetic, fluorescent beads were prepared as previously described and used to determine the levels of ADCD in the OPTIC and OCTAVE serum samples. The beads were diluted to a concentration of 50 beads/µl and 25 µl of the diluted beads was added to each well with 25 µl of HBSS and heat-inactivated serum at a final dilution of 1:100 or 1:500, conducted in duplicate. The beads and serum were incubated for 30 min at RT whilst shaking at 700 rpm, then washed twice in 100 µl of wash buffer (PBS containing 0.1% tween-20) and resuspended in 50 µl of HBSS with 10% IgG- and IgM-depleted human complement (Pel-Freeze Biologicals). The samples were incubated for 20 min at 37oC whilst shaking at 700 rpm, then washed twice in 100 µl of wash buffer and resuspended in 100 µl of FITC-conjugated C3c antibody (abcam) diluted 1:500 in HBSS. The samples were incubated for 20 min at RT whilst shaking at 700 rpm, washed twice in 100 µl of wash buffer, and resuspended in 50 µl of HBSS. Duplicate samples were combined and a minimum of 100 beads per sample were acquired on the BD LSRFortessa X-20 flow cytometer. The mean fluorescence intensity of FITC was determined using FlowJo (version 10) with the gating strategy shown in Supplementary Figure 2. MFI values were interpolated from a standard curve using 4-parameter logistic regression and then multiplied by the dilution factor. Standard curves were included in each experiment and the interpolated values were presented as arbitrary "complement activating units".

Instrument	BD LSRFortessa X-20 flow cytometer
Software	FlowJo Version 10
Cell population abundance	Not applicable - protein conjugated beads were used instead of cell populations.
Gating strategy	Fluorescent beads were first gated on using FSC-A and SSC-A. A second gate was then used to identify only the APC-fluorescent beads. Final statistics for mean fluorescence intensity were then determined for either PE or FITC.

☒ Tick this box to confirm that a figure exemplifying the gating strategy is provided in the Supplementary Information.
